# Periodic structures on liquid-phase smectic A, nematic and isotropic free surfaces

**DOI:** 10.3762/bjnano.9.34

**Published:** 2018-01-30

**Authors:** Anna N Bagdinova, Evgeny I Demikhov, Nataliya G Borisenko, Sergei M Tolokonnikov, Gennadii V Mishakov, Andrei V Sharkov

**Affiliations:** 1 Cryogenic department, P.N. Lebedev Physical Institute of the Russian Academy of Sciences, 53 Leninskiy Prospekt, Moscow, 119991, Russia; 2 Neutron Physics department, P.N. Lebedev Physical Institute of the Russian Academy of Sciences, 53 Leninskiy Prospekt, Moscow, 119991, Russia; 3 Federal scientific research centre “Crystallography and photonics” of Russian Academy of Sciences, 59 Leninskiy Prospekt, Moscow, 119333, Russia; 4 Division of Quantum Radiophysics, P.N. Lebedev Physical Institute of the Russian Academy of Sciences, 53 Leninskiy Prospekt, Moscow, 119991, Russia

**Keywords:** focal conic domains, free boundary, liquid crystals, microscopy, smectic A phase

## Abstract

The free boundary of smectic A (SmA), nematic and isotropic liquid phases were studied using a polarized optical microscope, an interferometric surface structure analyzer (ISSA), an atomic force microscope (AFM) and a scanning near-field optical microscope (SNOM). Images of the SmA phase free surface obtained by the polarized microscope and ISSA are in good correlation and show a well-known focal domain structure. The new periodic stripe structure was observed by scanning near-field optical microscopy on the surface of the smectic A, nematic and isotropic phases. The properties of this periodic structure are similar to the charged liquid helium surface and can be explained by nonlinear electrostatic instabilities previously described.

## Introduction

The considerable interest in studies of liquid crystalline free boundaries that has recently arisen is due to their intrinsic free surface properties which are not influenced by the substrate anchoring [1–2]. This is very important for many applications, such as display quality technology and production enhancement. The free surface of liquid crystals is interesting by itself because many applications use one single boundary (e.g., coatings, paints, make-up) [3–4]. Appreciable progress in the development of tools for surface characterization, such as interferometric surface structure analyzers (ISSAs, i.e., nanoprofilometer), atomic force microscope (AFM) [5–8] and a scanning near-field optical microscope (SNOM) [9–10] has been made.

To study the liquid crystalline free boundary structures, common nanotechnology tools are used, for example, AFM, light reflection, high-resolution microscopy, X-ray reflection, transmission electron microscopy (TEM), etc. We have applied the four most popular microscopy tools: polarized optical microscopy, ISSA, AFM, and SNOM and compared experimental images of focal conic domains (FCDs) [11–14] on the liquid crystalline free boundary delivered by these methods.

In our experiments, we have studied the LC compound 4-*n*-octyl 4'-cyanobiphenyl (K24 or 8CB), which has the following liquid crystalline phase sequence: isotropic (41 °C) nematic (32 °C) smectic A (22.2 °C) crystal phase.

## Results

**Figure 1:**
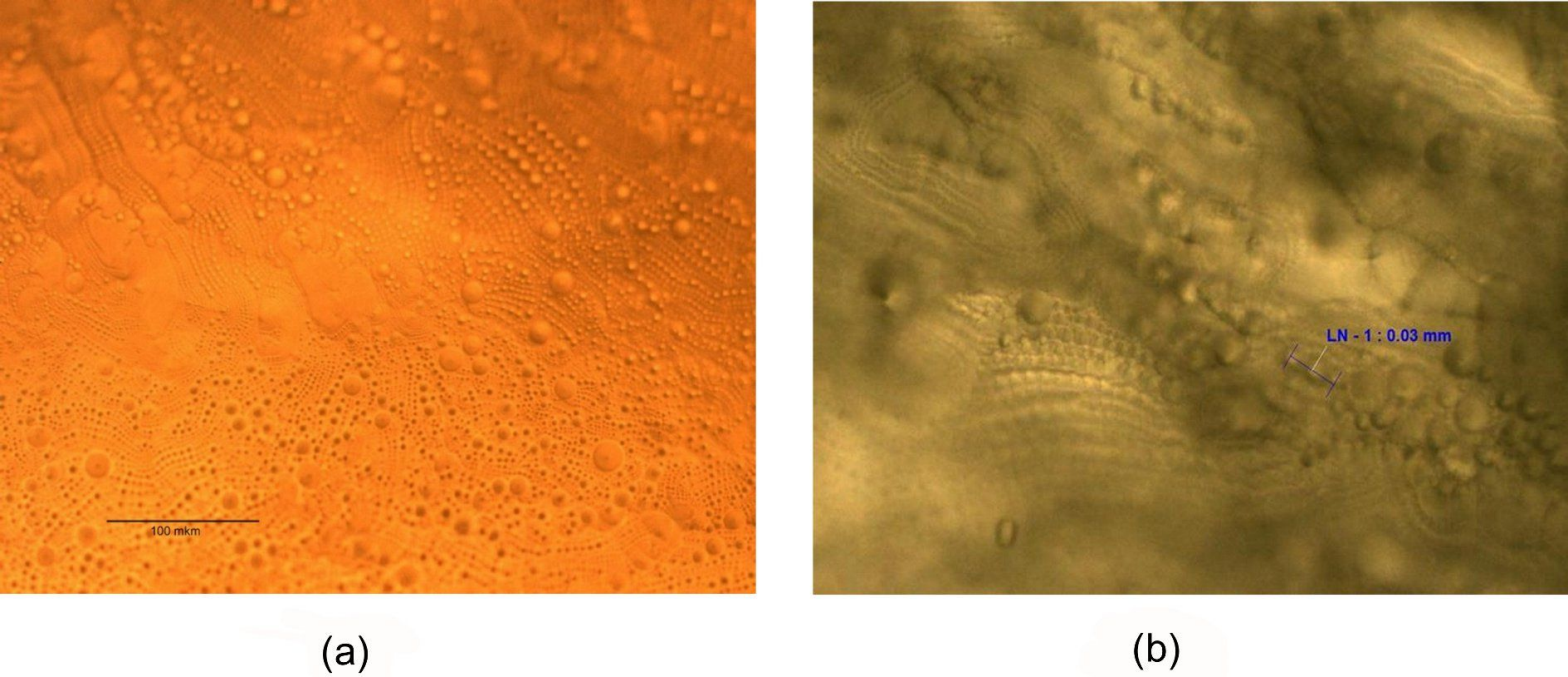
Image of the 8CB surface structures on the SmA free surface (a) and in bulk (b), as measured with an optical polarized microscope at 30 °C.

Figure 1 shows a surface structure of a SmA phase measured with a polarized optical microscope at 30 °C. The image presents crater and bubble-like structures formed on the SmA phase free surface and in the bulk of the substance as well. We see that these peculiarities are mostly ordered in chains on the surface and in the bulk. Comparison of our images with FCD images [15] shows great similarity with FCD domains observed earlier.

**Figure 2:**
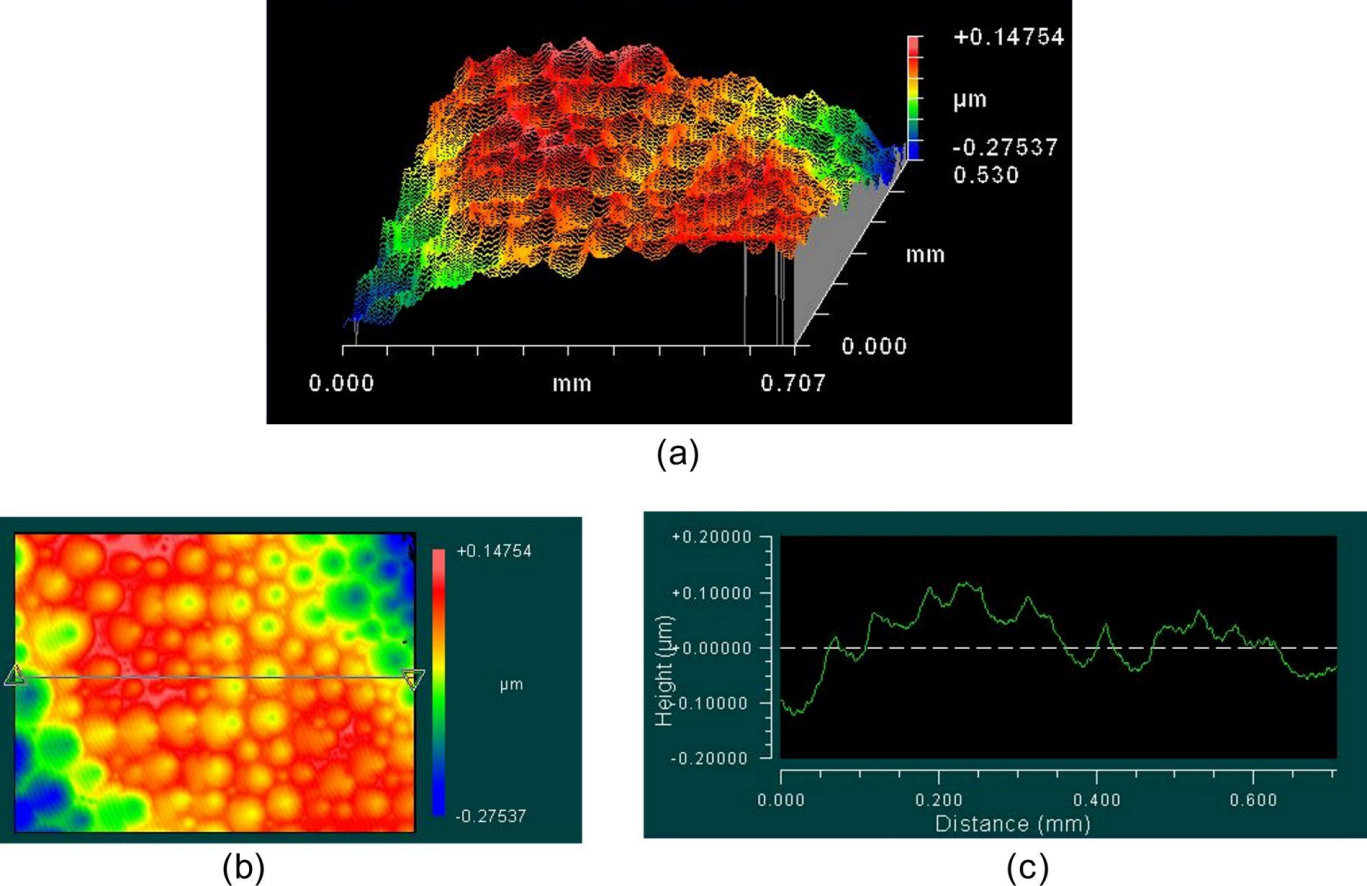
(a) Interferometric surface structure analyzer (ISSA) 3D reconstructed image of the SmA surface; (b) top view of Figure 2a; (c) cross-section profile on the line of the Figure 2b at *T* = 29 °C.

Figure 2 shows three-dimensional images of SmA surface structures obtained by the interferometric surface structure analyzer (ISSA). We see a system of craters and hills similar to the optical microscope image in Figure 1. An important feature of Figure 2 is the equidistant crater chains observed in different parts of the image. The range of peak-to-valley distances is 423 nm. The depth of the craters is 100 nm. With decreasing temperature, the craters become larger and deeper.

Experiments with different film thickness showed that the size of the crater-like structures became smaller as the film thickness decreased.

**Figure 3:**
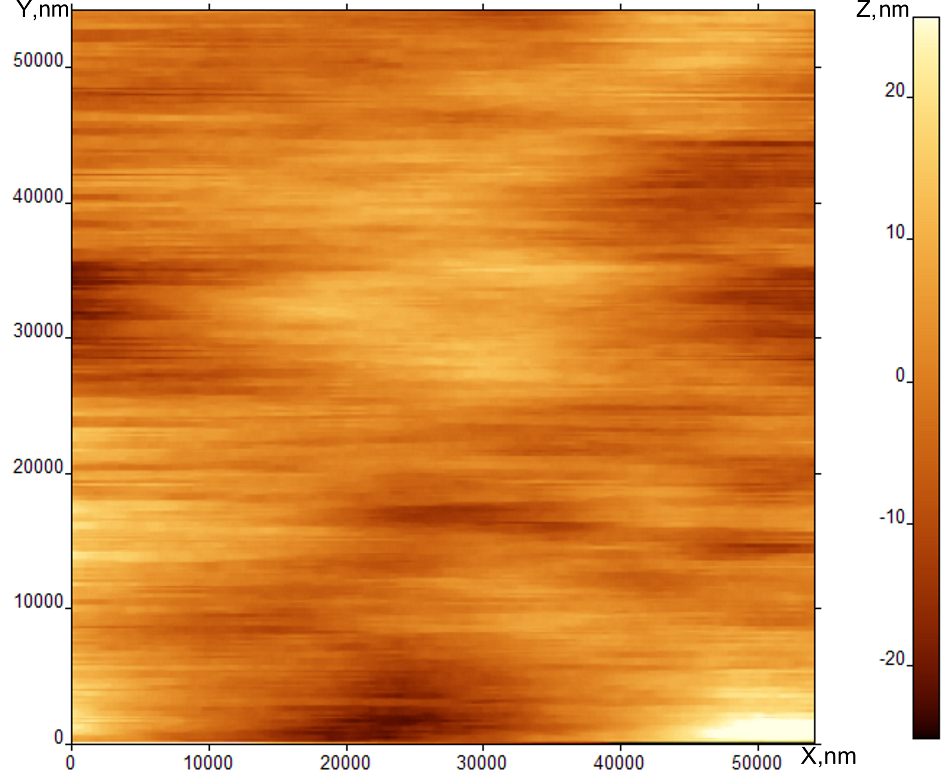
AFM image of SmA free surface at room temperature.

Figure 3 shows an AFM (topographical) scan of the SmA surface. The scratch-like character of the image shows that the tip of the probe microscope has directly contacted the SmA free surface and disturbs the surface structure during the scanning. The origin of this contact is not mechanical because the distance between the SmA surface and the tip is about 10 nm and is held constant during the scan. The interaction is likely Coulomb in nature.

**Figure 4:**
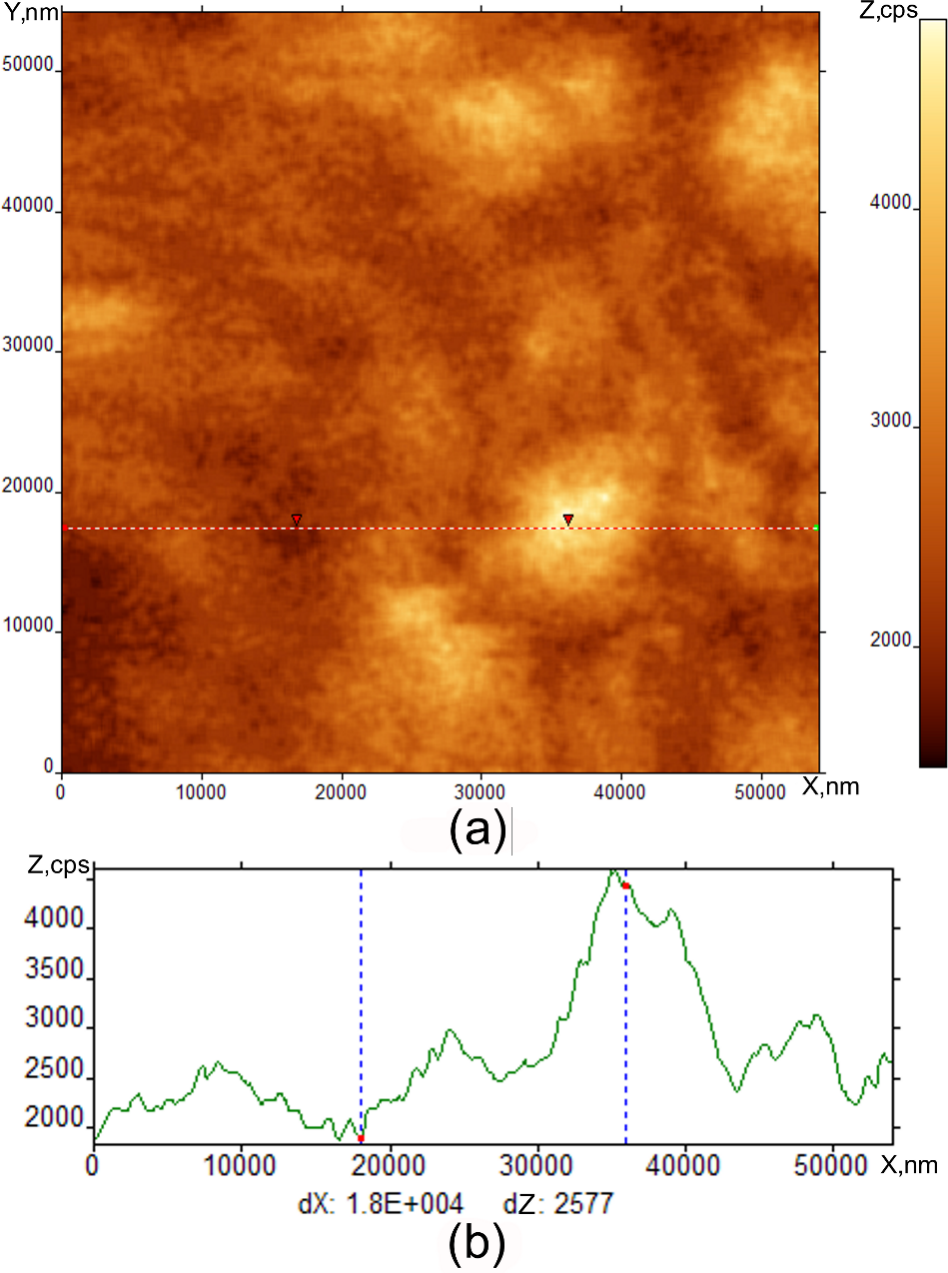
SNOM image of SmA free surface at room temperature (a) and its cross-section (b).

Figure 4 shows an image obtained with the SNOM and AFM profile of the surface. The intensity of the SNOM signal is measured in counts per second (cps). We see some irregular variation of contrast on the image. The size of this heterogeneity is about 10–15 μm. This value correlates within an order of magnitude with the size of the craters (FCD) observed with the ISSA in Figure 2. The distance between the tip and the surface was held about 20 nm and is larger than in Figure 4. The interaction between the tip and the surface is negligibly weak. This is the reason why we do not see the scratch-like perturbations due to the tip of the SNOM and instead some amorphous structures correlating with the FCDs.

**Figure 5:**
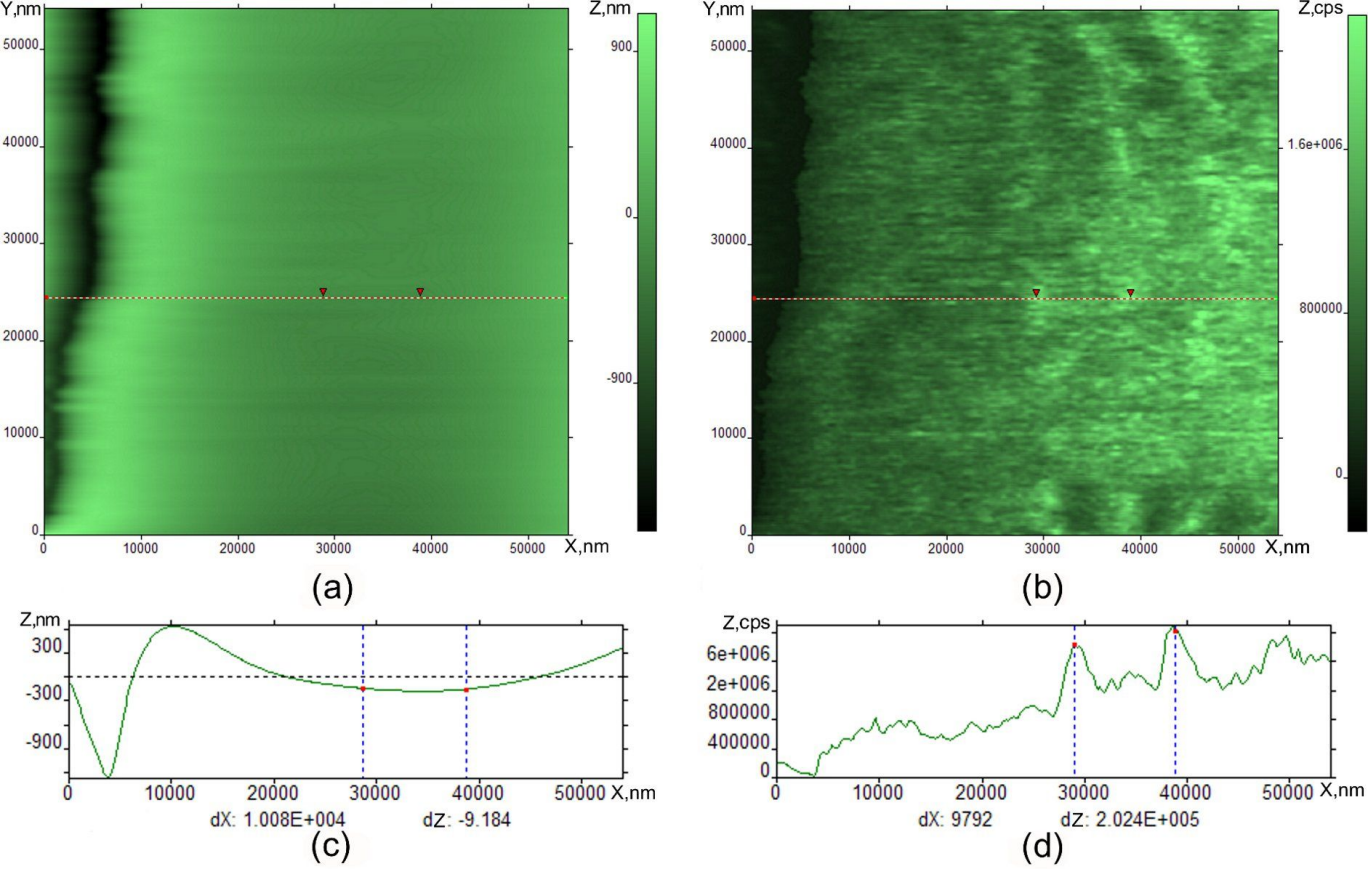
Periodic structure formation after Cry–SmA phase transition at room temperature. Topographical AFM (a) and SNOM (b) SmA droplet edge images on Si substrate, AFM and SNOM cross-sections (c) and (d).

In Figure 5, the tip is shifted to the position where we begin to see the interaction with the surface. This interaction is visualized in the AFM image in Figure 5a as scratch-like perturbations. The SNOM picture is completely changed in this case and we see the formation of periodic stripes perpendicular to the direction of motion of the tip.

**Figure 6:**
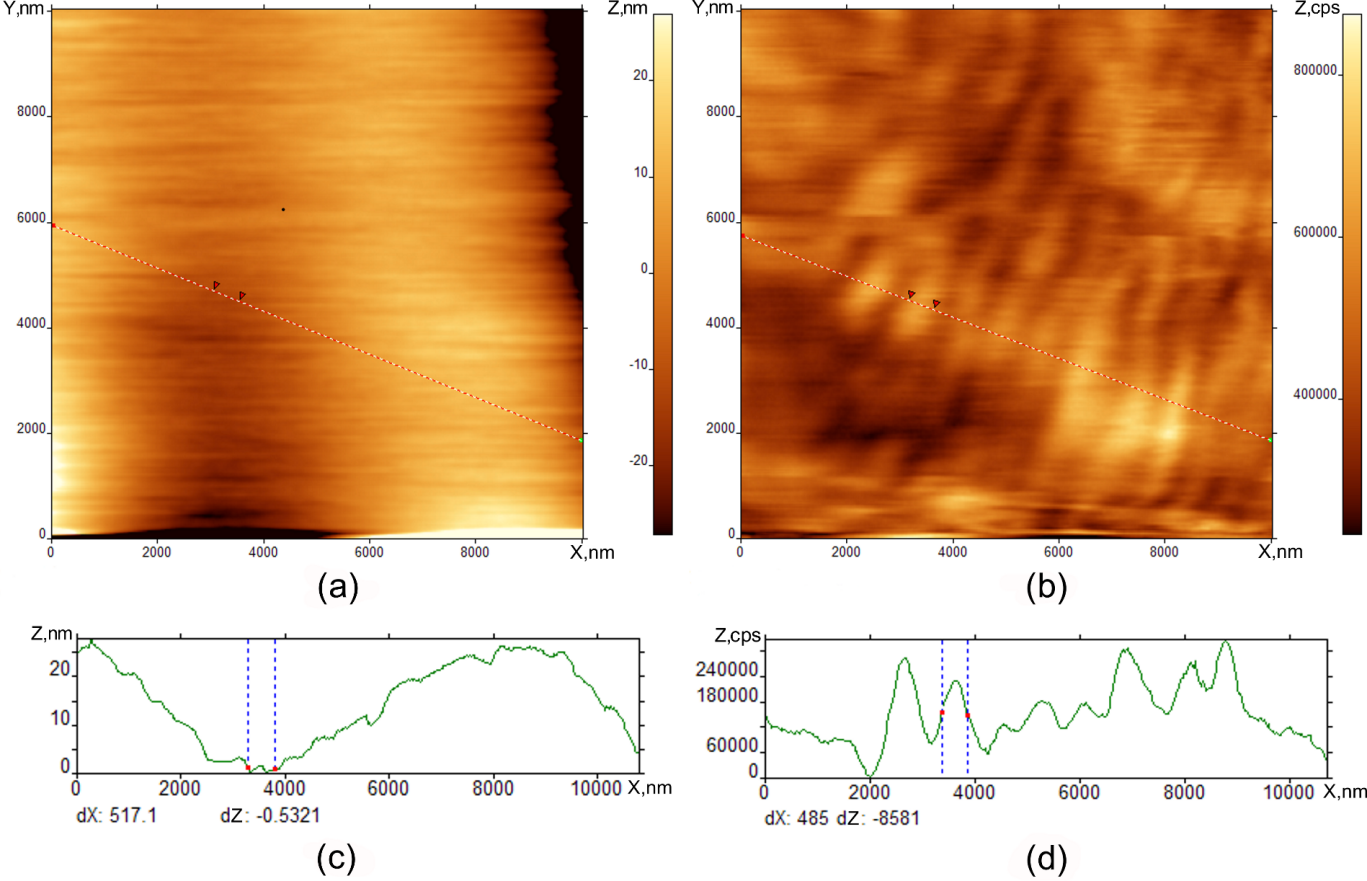
Periodic SmA structure formation at room temperature after cooling from the nematic phase. Topographical (a) and SNOM (b) images of SmA on Si substrate droplet edge, cross sections of both images (c) and (d).

Figure 6 shows the formation of the periodic stripe pattern in the SmA phase after the phase transition N–SmA. We observe a periodic structure in the SNOM image with a periodicity of 0.7–1 µm. The axis of the striped pattern is perpendicular to the tip motion.

**Figure 7:**
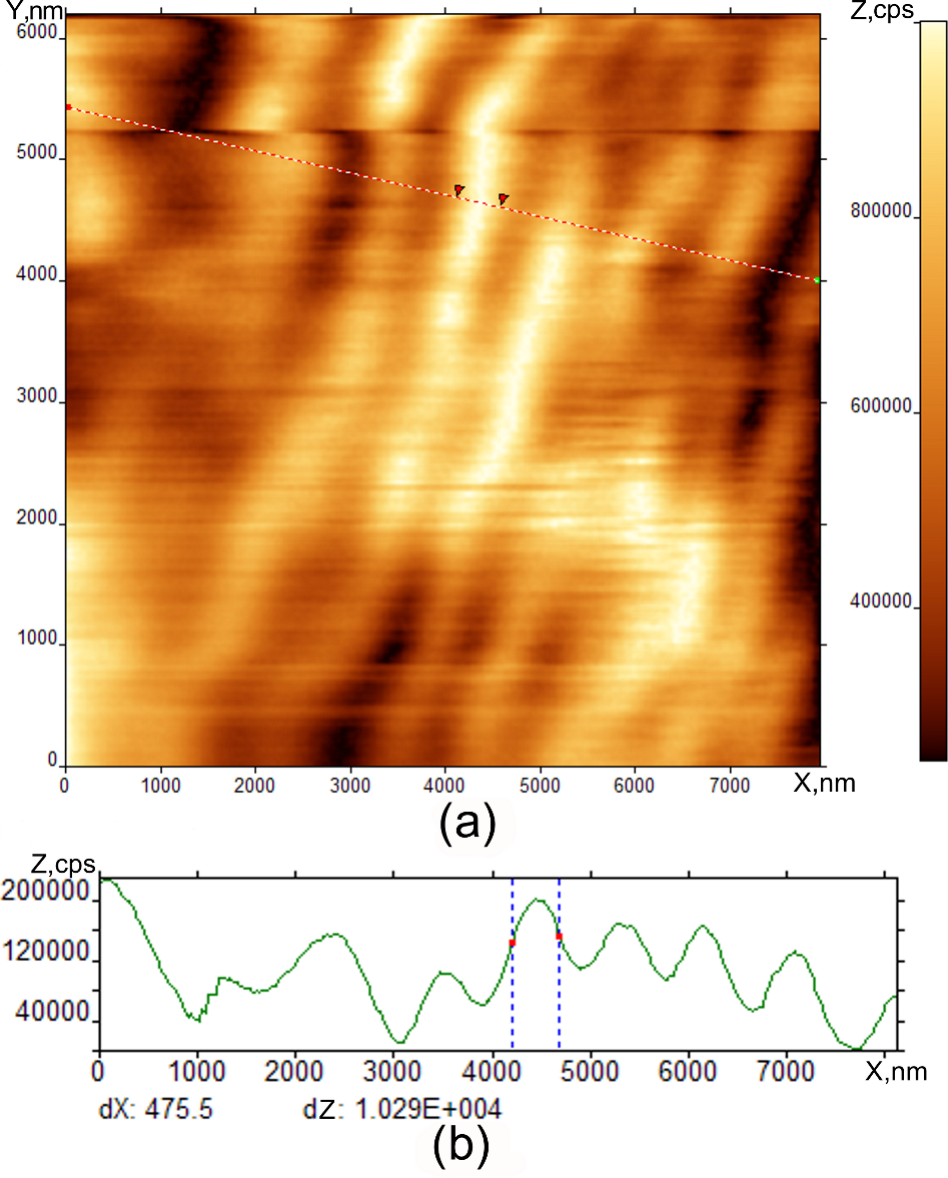
Equilibrium surface state of SmA phase after 30 minutes of relaxation at room temperature. SNOM image of SmA on Si substrate droplet edge at room temperature (a), optical image cross-section (b).

Figure 7 shows the evolution of the stripes with time. We see that the stripe periodicity becomes larger (Figure 5 and Figure 6) and the film thickness variation in the stripe region becomes smaller directly after the stripe formation. Generally, the stripe texture becomes smoother.

**Figure 8:**
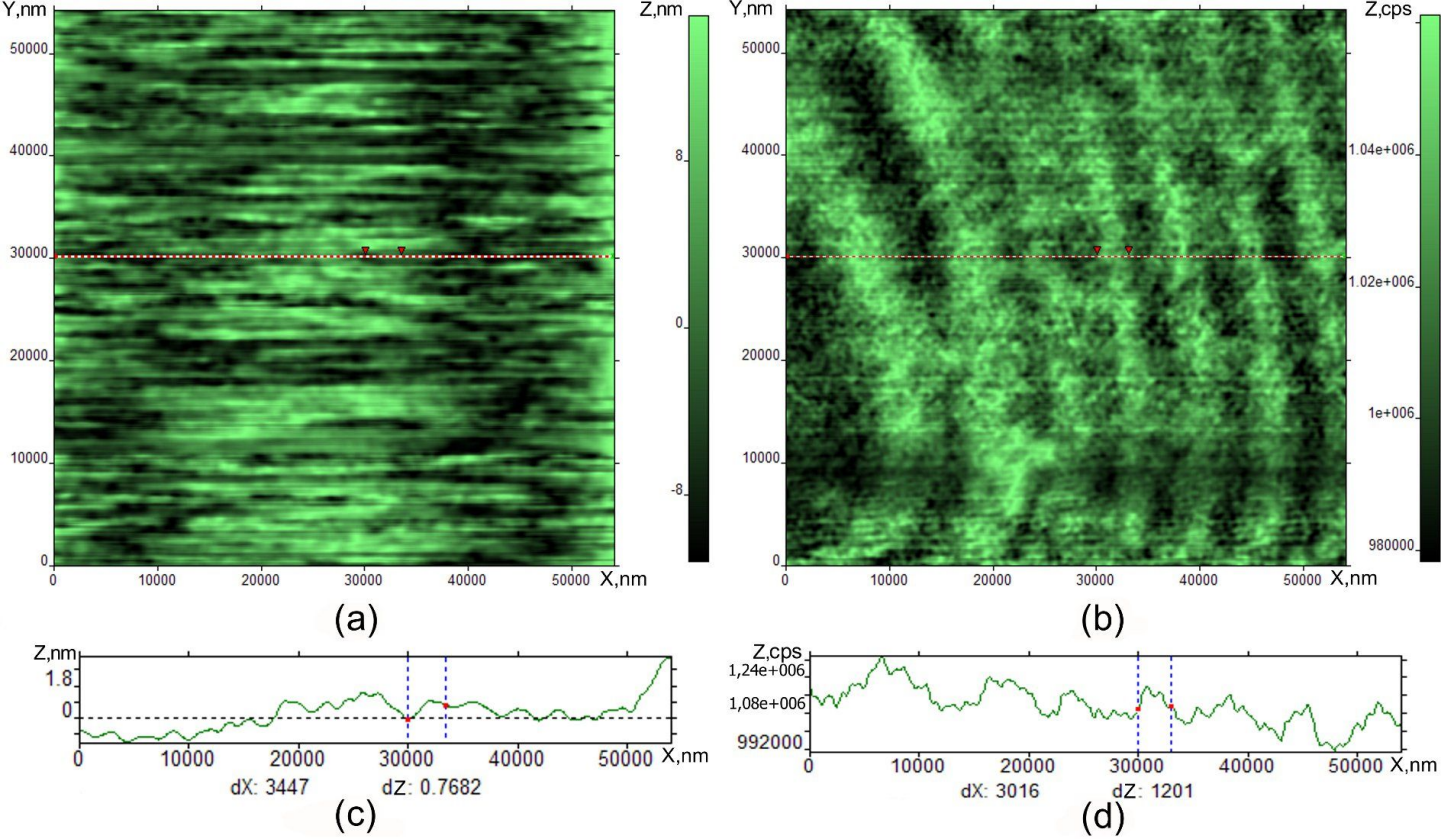
Periodic structure formation in the nematic phase at 38 °С. Topographical (a) and optical (b) SNOM image of SmA on Si substrate, cross-sections of both images (c) and (d).

Figure 8 shows the stripe formation on the free surface of the nematic phase at *T* = 38 °C. Directly after heating to 38 °С, the surface structure is homogeneous. Shortly after, a periodic striped structure perpendicular to the tip motion is formed on the nematic phase free boundary, similar to the SmA phase. The periodicity of stripes in the nematic phase is approximately ten times larger than in the SmA phase and the amplitude of film thickness modulation is ten times smaller than in the SmA phase at room temperature.

**Figure 9:**
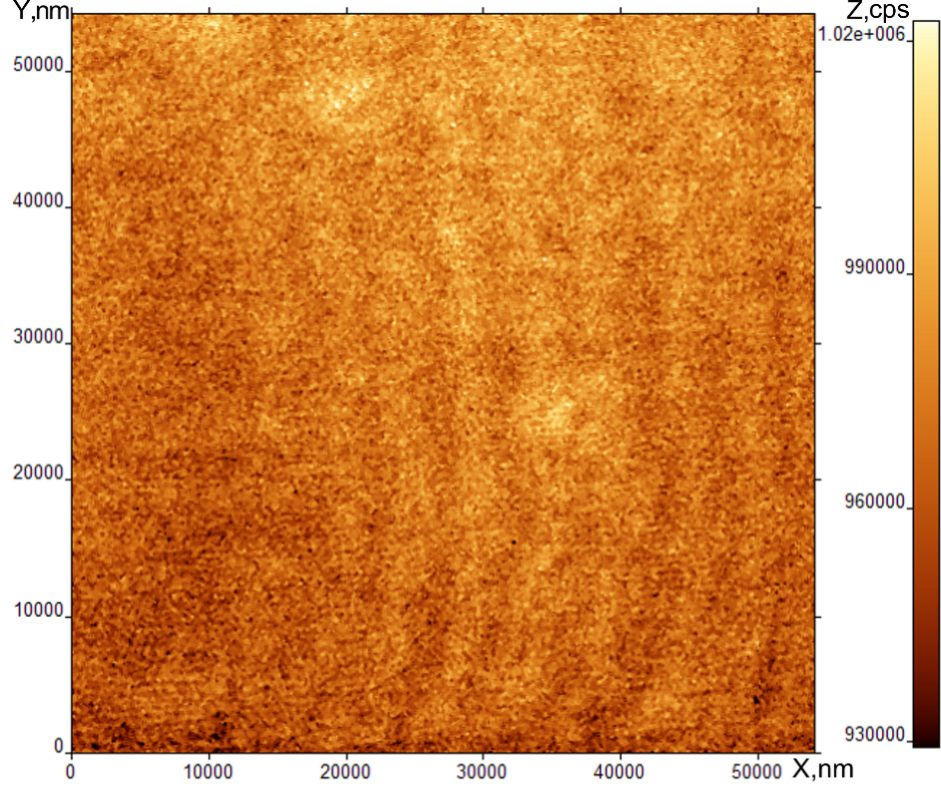
SNOM image of LC film on the Si substrate at 46 °С (isotropic liquid).

Figure 9 shows the stripe structure formation on the surface of the isotropic liquid, 8CB, at 46 °С. The contrast of this stripe texture is weaker than in the SmA phase. The stripe periodicity in the isotropic liquid is approximately the same as in the nematic phase.

## Discussion

**Figure 10:**
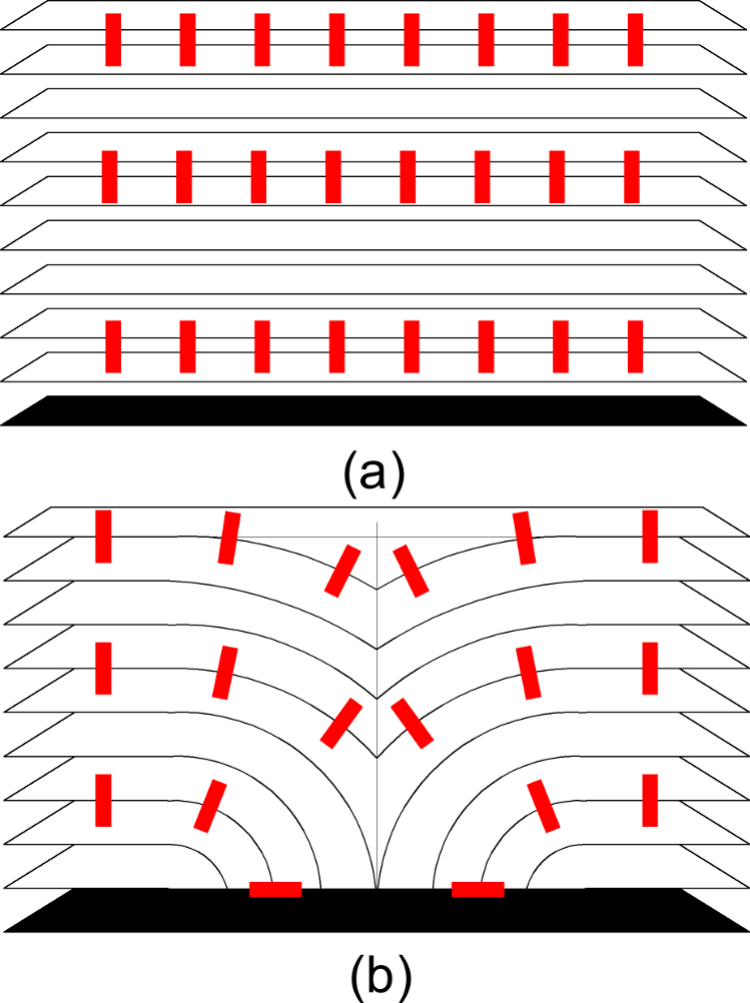
Schematic illustration of smectic layer configurations, with smectic layers (white), substrate (black), and representative rod-like molecules (red) (a,b). Smectic ground state (a). SmA toric focal conic domain (b) [15].

The comparison of optical microscopy and ISSA images with a previous paper [15] shows complete similarity with our results. Therefore, we can conclude that classical focal conic domains on the SmA phase free surface were observed. The model of the FCD structure is shown in Figure 10.

The FCDs are ordered in one-dimensional periodic chains which are shown in Figure 1 and Figure 2. The nature of this periodicity is elastic and is a property of the SmA phase because no FCDs are observed in the nematic phase and isotropic liquid. FCD images obtained by the ISSA and polarized microscope have similar parameters of structural peculiarities.

We observed a qualitative difference between the optical microscopy tools (ISSA and optical microscope) and probe microscopy images. The main feature is the observation of periodic stripes perpendicular to the scan direction in the case of the interaction between the tip and the surface. It should be noted that the striped pattern in Figure 7a, Figure 8b, and Figure 9 is not a simple interference picture, confirmed by the observation of a periodic surface valley and tip profile on the same sample. The presence of the interaction between the tip and surface is indicated by scratches in the AFM and SNOM images. When the tip is far from the surface in Figure 4, we observed some structure-less irregularities whose dimension correlates to the FCD dimensions in Figure 1 and Figure 2. When the tip comes closer to the surface, the pattern is completely changed, and stripes are seen by SNOM. The striped formation and its properties can be explained by some combination of reasons including Coulomb interaction combined with standing surface wave formation. The stripes are induced and observed in one and the same scan by SNOM. In the SmA phase, the stripes are deeper and possess smaller periodicity than in the nematic and isotropic liquid. The mechanical formation of surface standing waves can be the initial mechanism of stripe appearance. The mechanism of stripe formation can be the following. We bring the tip in position where the interaction with the surface occurs. The tip motion gives rise to a surface wave formation, like a standing wave on a water surface. By the same tip, motion surface charges are induced. Therefore, we have charged the LC surface which is influenced by some periodic tip motion. This case is similar to the problem of nonlinear periodic instabilities on a charged liquid surface [16–20]. In the article [16], a case was considered where periodic stripe structures occur on the charged liquid helium surface as a result of competition between electrostatic forces and surface tension. Qualitatively, our problem is similar to this case and can be described by similar equations. This mechanism explains why several minutes are needed to form the stripes. This time corresponds to charge diffusion to the stable position in the striped structure. This mechanism explains the striped appearance in the nematic phase and isotropic liquid. After some relaxation time in the SmA phase, the stripes become smoother and the periodicity larger. Because of the layered structure of the SmA phase, the energy barrier exits for the surface reconstruction, therefore the stripes are more pronounced in the SmA phase. The observation of stripes in the nematic phase and isotropic liquid shows the independence of the stripe nature on the liquid crystal phase properties. The stripes disappear with increasing temperature due to the disappearance of the elastic constants on the surface. The striped formation is not a surface ordering effect like in [2,16]. On the surface ordering [2,21–24], the surface remains smooth and we observe only director orientation variation.

## Conclusion

A complex study of the liquid crystal 8CB free surface in the smectic A phase was carried out. The structures of the free surface are studied using four techniques: Polarized microscopy, ISSA, SNOM, AFM. The applicability of surface-sensitive tools for liquid crystals was reviewed. We observed a periodic order of FCDs in one-dimensional chains. A periodic stripe texture is observed in SmA, nematic phases and isotropic liquid in 8CB. The periodic images of the probe depend on the distance between the tip and the material. The periodic surface waves appear due Coulomb repulsion forces and standing surface wave’s formation on the LC surface.

## Experimental

The studied material, 8CB (4-*n*-octyl-4’-cyanobiphenyl), has the following liquid crystalline phase sequence: isotropic (41 °C), nematic (32 °C), smectic A (22.2 °C) crystal phase.

**Figure 11:**
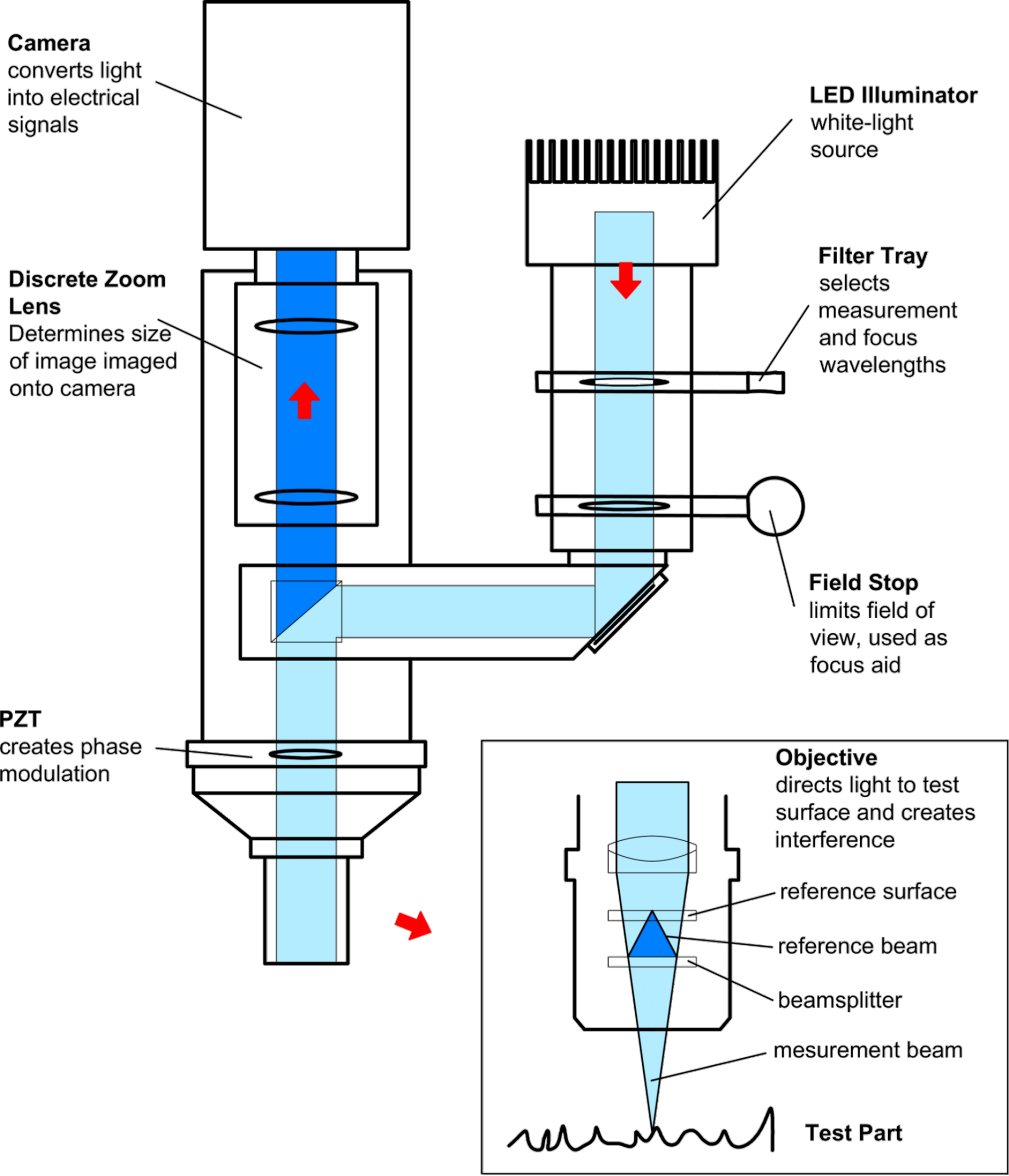
Schematic optical diagram of the interferometric surface structure analyzer (ISSA).

As a first step, we studied the free boundary of 8CB with a nanoprofilometer (ZYGO NewView 6K). A schematic diagram of the ISSA is shown in Figure 11. Scanning interferometry of white light is used in the ISSA to obtain images, calculate, and analyze the surface structure of samples. Light from the microscope is split in the interferometric objective; one part of the light beam reflects from the sample and another reflects from the internal reference surface in the objective. Both parts are then directed to the solid-state camera.

The result of the interference of the two wavefronts is an image of light and dark fringes that indicate the presence of the surface structure. The sample beam scans in vertical movements of the objective with a piezoelectric transducer (PZT). Intensities of each camera pixel are recorded with a video camera and converted to amplitudes by the program MetroPro.

**Figure 12:**
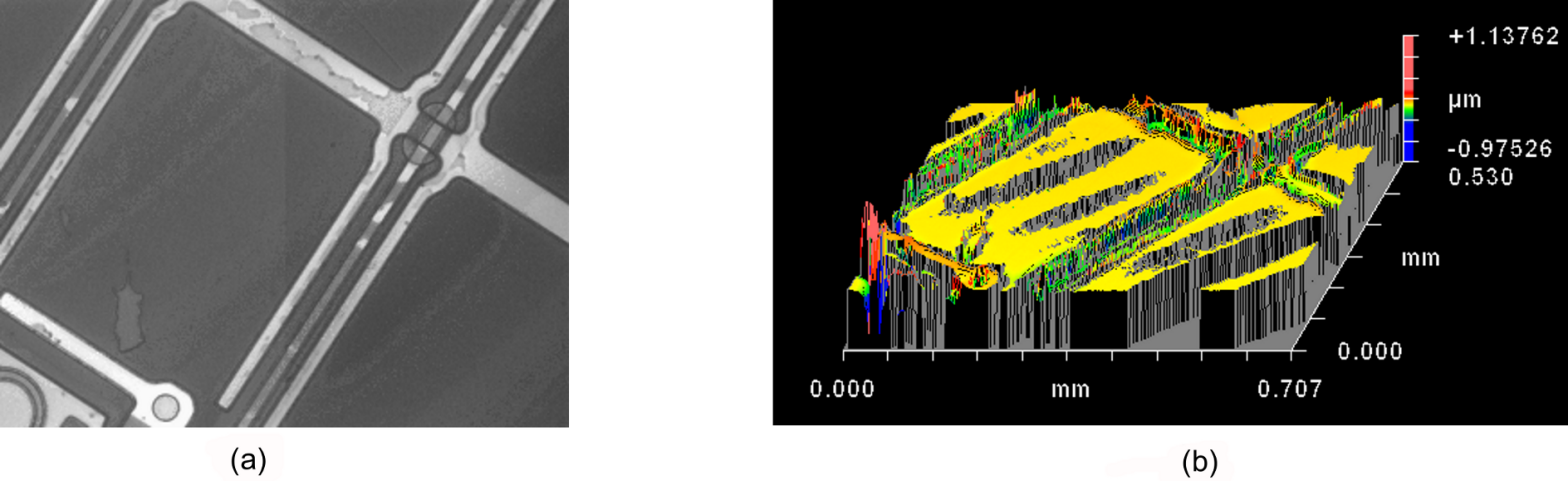
Liquid crystalline display substrate a) without a material; b) a 3D reconstruction of the display with a very thin LC film.

A piece of LC display was used as a substrate (Figure 12), which provides two main advantages. First, it has a large anchoring energy for theliquid crystal director field, and second, helps to better focus at the bottom edge of the liquid crystal layer.

During the experiments, a small droplet of the substance was placed on the display substrate with a sharp metallic tip and then put on the heating stage. The temperature stability was about 0.1 °С. The temperature gradient was measured by the SmA–nematic phase boundary appearance and motion and was about 0.1 °С. The temperature of the isotropic liquid phase was set, and the behavior of the droplet was studied using ISSA.

In some experiments, the droplet was thinly spread on the substrate with a metal blade in the isotropic liquid. To measure the liquid crystal layer thickness, the ISSA was focused on the upper boundary LC–air interface and after that on the boundary of the LC–solid substrate (the transparency of the object made this possible). The difference between these microscope positions gives the thickness of the object.

The next interesting and relatively new tool for surface study of LCs is the scanning near-field optical microscope (SNOM). The SNOM has a sharpened optical fiber probe with an aperture much smaller than the wavelength. The probe detects (or excites) the so-called "evanescent" ("vanishing") component of the alternating electric field of the optical signal, which rapidly decays at short distances from the source. In this case, the spatial resolution is not limited to the diffraction limit of the classical optical microscope and is determined by the size of the aperture at which the optical signal can still be recorded.

**Figure 13:**
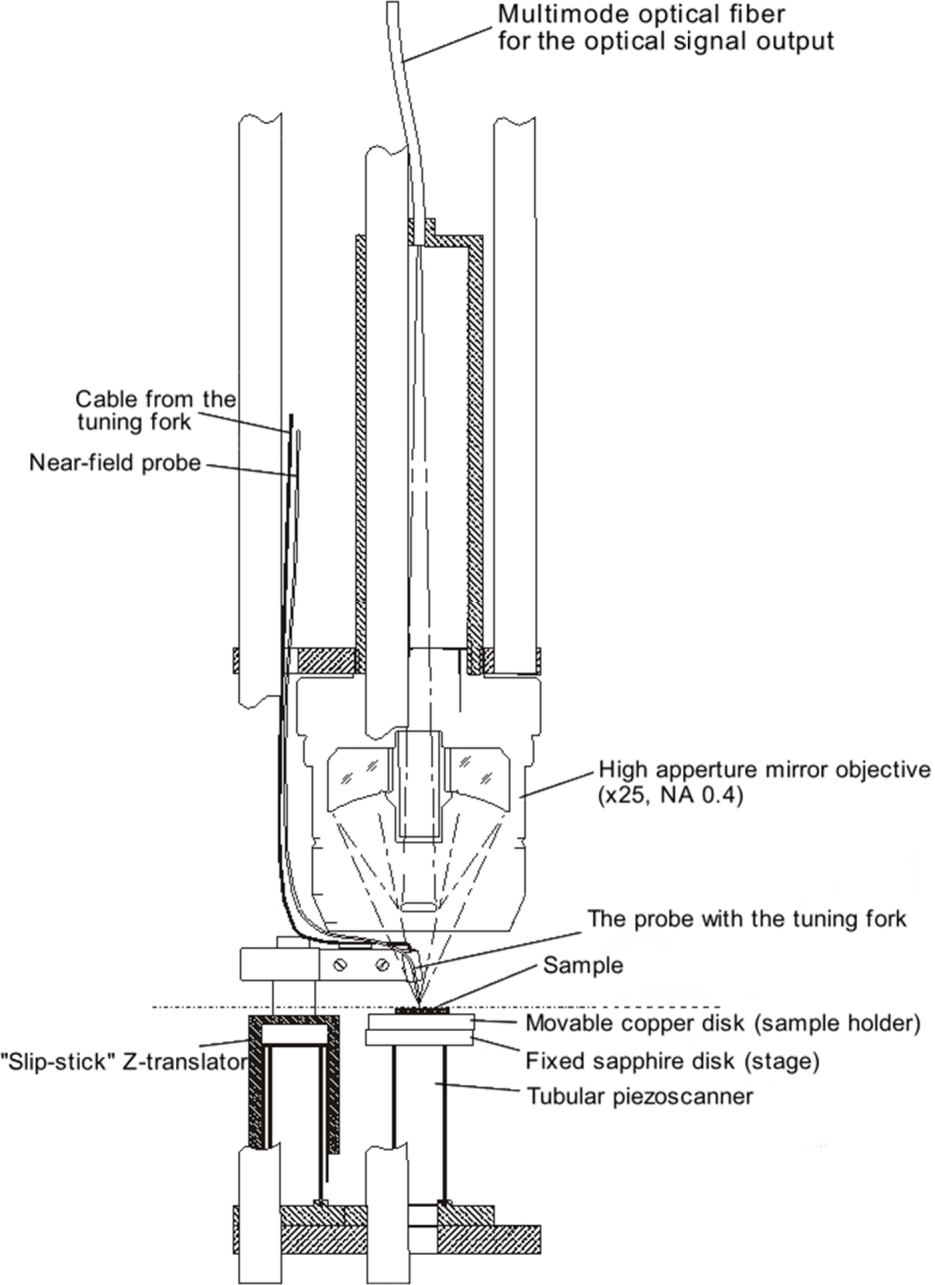
Schematic diagram of the CryoSNOM produced by CDP System Corp. (http://www.cdpsystems.com/moscan.html).

The optical scheme of the SNOM is shown in Figure 13. A laser of wavelength of 532 nm was integrated into the probe. The reflected light from the sample was collected by a mirror lens and directed along a multimode fiber to the photomultiplier. The magnitude of the optical signal was measured in the photon counting mode. We used two modes of detection: the first is known as the topographical mode, which is similar to AFM detection. In this case, the tip levitates over the surface without any direct connection at a fixed distance to the sample. The optical SNOM mode gives an optical image which is obtained through a 200 nm diameter hole in the tip. In this case, the tip is in direct contact with the surface but is not immersed in the LC surface. Generally, the distance between the tip and the surface can be changed in the interval of 0–20 nm using a piezoelectric shifter. For optical microscopy, we used a versatile polarized optical microscope (HUVITZ HRV-300) which has polarized and confocal modes.

**Figure 14:**
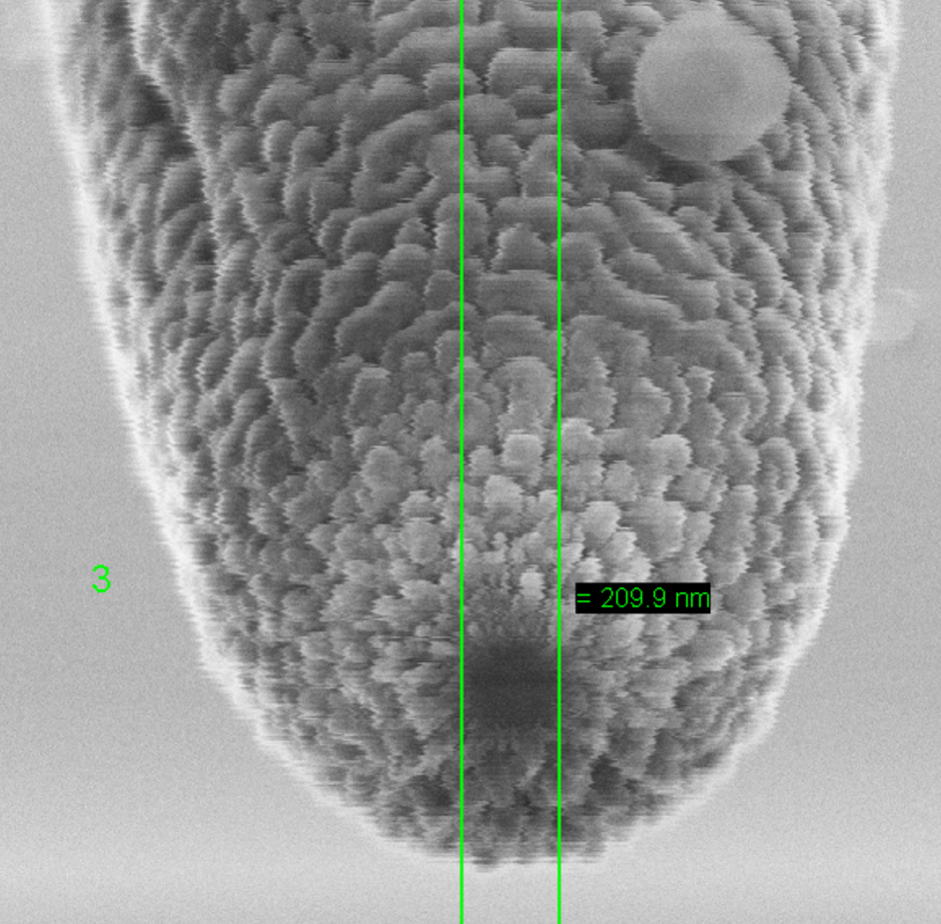
SEM photo of the SNOM probe in the topographical regime, where the scales represent Al metallization. (Photo provided by V. V. Rogov, IPM RAS).

Figure 14 demonstrates a scanning electron microscope photo of the SNOM probe with a magnification of about 104, extra high tension of 10 kV, tilt angle of 0°, and an aperture size of 30 μm. The aperture is about 200 nm.

**Figure 15:**
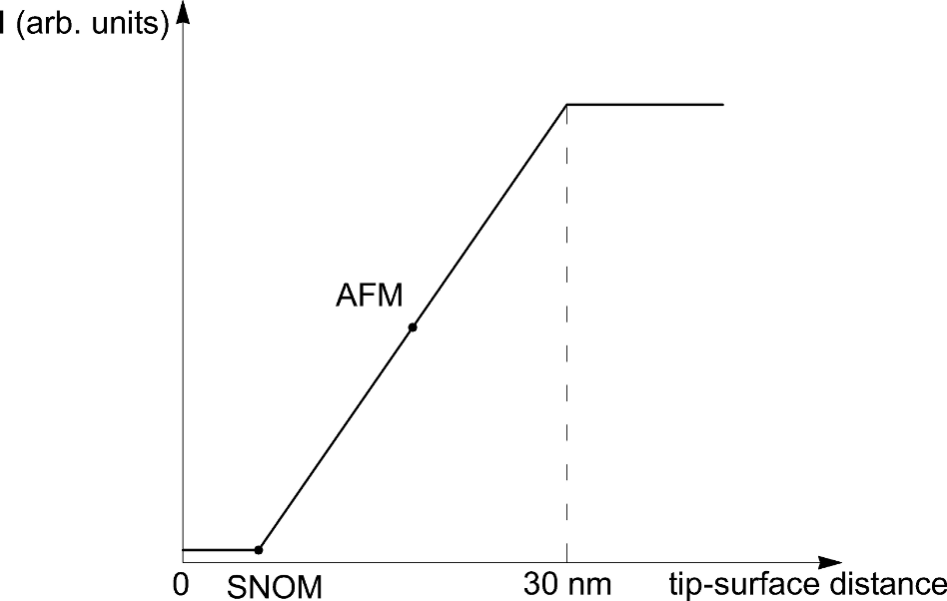
Schematic illustration of operational regions of SNOM and AFM.

Figure 15 shows the regions of operation of SNOM and AFM. These regions can be defined in the following way. We start to move the tip from the infinity to the surface. The optical signal from the photomultiplier is constant until a small distance of about 30 nm, where it starts to diminish. The signal decreases to zero which corresponds to the connection of the tip and the surface. Usually, this is the operation point of SNOM. The AFM operation point is in the middle between two saturation regions. The typical distance between the tip and the surface is 10–20 nm. In our case, we register SNOM and AFM pictures at the same time. Our operational region corresponded to a 10 nm distance between the tip and the surface but can be shifted to other values. The interaction between the tip and surface begins at a distance of 30 nm.
